# Preverbal Production and Early Lexical Development in Children With Cochlear Implants: A Longitudinal Study Following Pre-implanted Children Until 12 Months After Cochlear Implant Activation

**DOI:** 10.3389/fpsyg.2020.591584

**Published:** 2020-11-19

**Authors:** Marinella Majorano, Margherita Brondino, Marika Morelli, Rachele Ferrari, Manuela Lavelli, Letizia Guerzoni, Domenico Cuda, Valentina Persici

**Affiliations:** ^1^Department of Human Sciences, University of Verona, Verona, Italy; ^2^U.O. Otorhinolaryngology, Guglielmo da Saliceto Hospital, Piacenza, Italy

**Keywords:** Cochlear-implant, children, babbling, first words, latent growth analysis, language development

## Abstract

Studies have shown that children vary in the trajectories of their language development after cochlear implant (CI) activation. The aim of the present study is to assess the preverbal and lexical development of a group of 20 Italian-speaking children observed longitudinally before CI activation and at three, 6 and 12 months after CI surgery (mean age at the first session: 17.5 months; SD: 8.3; and range: 10–35). The group of children with CIs (G-CI) was compared with two groups of normally-hearing (NH) children, one age-matched (G-NHA; mean age at the first session: 17.4 months; SD: 8.0; and range: 10–34) and one language-matched (G-NHL; *n* = 20; mean age at the first session: 11.2 months; SD: 0.4; and range: 11–12). The spontaneous interactions between children and their mothers during free-play were transcribed. Preverbal babbling production and first words were considered for each child. Data analysis showed significant differences in babbling and word production between groups, with a lower production of words in children with CIs compared to the G-NHA group and a higher production of babbling compared to the G-NHL children. Word production 1 year after activation was significantly lower for the children with CIs than for language-matched children only when maternal education was controlled for. Furthermore, latent class growth analysis showed that children with CIs belonged mainly to classes that exhibited a low level of initial production but also progressive increases over time. Babbling production had a statistically significant effect on lexical growth but not on class membership, and only for groups showing slower and constant increases. Results highlight the importance of preverbal vocal patterns for later lexical development and may support families and speech therapists in the early identification of risk and protective factors for language delay in children with CIs.

## Introduction

Several studies have shown that early language emerges from the interaction of several components, such as phono-articulatory skills and perceptual abilities, and that its development involves both common patterns and individual trajectories (Kuhl and Meltzoff, 1982; Hoff, 2013; Kuhl et al., 2014; Vihman, 2014). In fact, despite a certain degree of variability in onset time and characteristics across children (Keren-Portnoy et al., 2009), observational studies have shown that most children follow the same pattern of early vocal development, going from vocalizations (around 1–4 months) to babbling (around 5–10 months) and, finally, to producing first words around 10 months of age (Menyuk et al., 1986; Stoel-Gammon, 1989, 2011; Whitehurst et al., 1991; Ferguson et al., 1992; Kuhl et al., 2008). It has been suggested that preverbal vocal patterns have an important developmental function: they are thought to allow children to develop a link between articulatory settings and auditory consequences, and to lay the foundations for the development of a phonetic inventory and for adapting language output to environmental input (Westermann and Miranda, 2004; Vihman, 2014).

According to several authors (Stoel-Gammon, 2011; Vihman, 2014), babbling (whether canonical – when containing one consonant and one vowel – or variegated – when containing different vocalic and consonantal elements; Vihman, 2014) is especially important for the construction of the early lexicon for two main reasons. First, it allows children to construct “sensorimotor representations” (Davis and MacNeilage, 2000; Lindblom, 2000; Locke, 2001) with the support of an “auditory feedback loop” (Fry, 1966, p. 189) based on implicit or procedural learning (Squire and Zola, 1996). Second, a child’s babble contains recognizable linguistic sounds (McGillion et al., 2017): this makes the caregiver more responsive to the child’s initiatives than to other types of simple vocal production, thus creating more opportunities for interactions that resemble “proto-conversations” (Bloom et al., 1987; Gros-Louis et al., 2006) and more opportunities for the child to learn the phonological patterns of the adult target words (Goldstein and Schwade, 2008). By virtue of their greater articulatory experience (Majorano et al., 2014) and adult speech exposure, children who are at a more advanced stage of preverbal production should show more advanced lexical development later on. In line with this idea, observational studies have found an association between preverbal production (in particular babbling) and early words-production in children with typical development (Oller et al., 1999; Majorano et al., 2014; McGillion et al., 2017) and in children with language delay (Rescorla et al., 2000; Keren-Portnoy et al., 2009).

The link between preverbal and verbal production is likely to cause a set of negative consequences for the spoken lexical development of children with hearing loss, who are deprived of early auditory experiences. A study by Kishon-Rabin and colleagues (2005), conducted on 24 children with unilateral hearing loss by means of a self-report parenting questionnaire, has shown that children with hearing loss do show delayed onset of preverbal vocalizations relative to their normally-hearing (NH) peers. This delay suggests that onset of babbling production is not only constrained by anatomical and physiological factors, but also by auditory perception and phono-articulatory experience.

To successfully develop communication abilities, children with hearing loss have two possibilities. One alternative is for children to acquire sign language, a fully-fledged, natural language with a similar developmental trajectory to that of spoken language, with acquisition of manual babbling taking place between 6 and 12 months and first word emergence occurring around 10 months of age (see Mayberry and Squires, 2006). The other alternative is for them to gain access to spoken language input through medical devices such as cochlear implants (CIs). CIs are devices that can be surgically implanted and that are able to provide access to auditory information to children with severe to profound deafness by electrically stimulating their auditory nerve; despite having some surgery-related risks, cochlear implantation is mostly regarded as beneficial to children with hearing loss and considered as a relevant alternative to sign language acquisition, as it constitutes a viable option for oral language development despite the absence of early exposure to speech sounds (Geers, 2006). Observational studies have shown that, after the activation of the implant, both vocalization, and canonical babbling emerge (Walker and Bass-Ringdahl, 2008; Schramm et al., 2010). As shown by Löfkvist et al. (2019) in a pilot study with a small sample of children with CIs and hearing aids, the activation of the implant appears to cause a “trigger effect” on canonical babbling onset. According to Schauwers and colleagues (2004), children who are implanted between 6 and 18 months of age begin babbling within 4 months of language exposure, similarly to age-matched peers. However, the reduced early phono-articulatory experience and their limited opportunities for matching phonological patterns to speech input may still cause negative effects on their preverbal production. In fact, their limited phonetic inventory before implantation is thought to reduce their sound combination possibilities in variegated babbling (McDaniel and Gifford, 2020). In addition, it is unclear whether babbling production in this population follows the same developmental pattern as in NH children (Moore and Bass-Ringdahl, 2002; Schramm et al., 2010). The preverbal competences of children with CIs appear to have a significant degree of within-group variability in their growth rate (Ertmer and Mellon, 2001; Ertmer et al., 2002): some children with CIs catch up and reach the babbling levels of NH children, while others show a slower evolution of preverbal skills (Schramm et al., 2010). As shown in a recent systematic review by McDaniel and Gifford (2020), most studies investigating prelinguistic vocal development of children with cochlear implants (with an average sample size of eight participants and mostly focusing on canonical babbling) have reported a lower production of preverbal utterances before CI activation, but also a higher growth of syllable production after cochlear implantation with respect to NH children with the same hearing age but lower chronological age. The older chronological age of the children with CIs compared, and consequently, the greater physical, cognitive, and social maturity associated with it, could explain their rapidity in babbling growth compared to younger normally hearing children that are matched for hearing age (Ertmer and Inniger, 2009).

Understanding preverbal developmental patterns in this population is important, because deficits in preverbal production can have repercussions on the children’s lexical development as well. In fact, connections between preverbal and verbal development have also been found in children with hearing loss and CIs (Oller and Eilers, 1988; Schauwers et al., 2004; Kishon-Rabin et al., 2005; Walker and Bass-Ringdahl, 2008). In a recent study, Jung and Houston (2020) showed that children with CIs also show associations between preverbal and verbal production, and, more specifically, between onset timing of babbling and speech recognition and vocabulary size 24 months after implant activation. In line with other authors, they interpreted their findings by claiming that onset time of babbling production is relevant because children develop perceptual skills not only by listening to others, but also by listening to their own verbal production. Given the association between onset time of preverbal production and lexical development, the time delay shown by the children with CIs (especially relative to utterances containing consonant sounds) with respect to their age-matched peers is likely to cause an additional, negative effect on their language development, on top of the issues related to their inability to hear speech input.

In line with this idea, research has shown that the early lexical production of children with CIs appears to be delayed (Svirsky et al., 2000). However, results are not completely consistent across studies, also due to high variability in sample characteristics and individual profiles (Spencer, 2004), including at what age children were diagnosed (Apuzzo and Yoshinaga-Itano, 1995) and implanted (Dettman et al., 2007; Nicholas and Geers, 2007; Nott et al., 2009a, b) and whether or not children were exposed to sign language before implantation (see Davidson et al., 2014, and Geers et al., 2017). Early language differences also seem to play a role: for example, in a longitudinal study, Szagun and Schramm (2016) have shown that the variability in the language development of children with CIs between 24 and 30 months after CI activation was predicted by individual differences in early vocabulary growth (an early indicator of individual differences in the children’s ability to learn). Attention should also be paid to factors not depending directly on the child, like socio-economic status and maternal education, which have been shown to affect maternal language input and, as a consequence, child language development as well (Hoff, 2006; DesJardin et al., 2014).

In sum, neither the trajectories of preverbal and lexical development, from before implantation to after CI activation, nor the contribution of early preverbal expressive skills to later language outcomes in children with CIs are clear. It is also unclear whether and how these differ between CI and NH children. According to observational studies, babbling production in children with CIs may be an expression of the emerging connection between their developing receptive abilities and their articulatory (a connection aimed at building phonological representations), and its practice may particularly support language acquisition in this group.

The present study aims to investigate the trajectories of preverbal production and first words and the role of preverbal production in affecting lexical growth by looking at three groups of children, one of children with CIs and two of NH children, one matched for chronological age (G-NHA) and the other matched for language level (G-NHL), in interaction with their mothers. The study adopts a longitudinal approach that starts before CI activation and includes assessments at 3, 6, and 12 months after it. An additional value of the present study is that it provides data on these aspects in a language other than English, which is important for understanding whether findings are language-specific or generalizable; moreover, no study, to the best of our knowledge, has reported longitudinal comparisons between children with CIs and normally hearing children acquiring Italian. The specific aims are:

1. To compare the development of babbling production in CI children (younger than 3 years) with that of both age-matched and language-matched children, before surgery and in the first year after CI activation (Stoel-Gammon, 2011). In accordance with previous studies (McDaniel and Gifford, 2020), we expected that: (i) the group of CI children would have slower preverbal development as compared to the G-NHA group, and similar development to the G-NHL children; (ii) the children with CIs would recover the delay within 6 months after CI activation, albeit with individual differences.
2. To compare the lexical trajectories of the group of children with CIs (G-CI) with those of the G-NHA and G-NHL children, before surgery (T1) and at three (T2), six (T3), and 12 (T4) months after CI activation. We expected that the G-CI children would display a delay in lexical development. Specifically, the hypotheses were that: (i) the lexical skills of the G-CI children would improve significantly after activation, (ii) the language development of the G-CI children would be slower than that of the G-NHA children but similar to that of the G-NHL children; and (iii) the delay of the CI children would reduce between the first and the third session after CI activation.
3. To compare children with CIs with children with NH with regard to the relationship between babbling production and lexical development. We expect babbling to affect lexical growth, especially in children with less steep growth rate curves.

## Materials and Methods

### Participants

Twenty dyads of hearing mothers and children with hearing loss (11 males and 9 females) were recruited from the “Guglielmo da Saliceto” hospital (G-CI); forty dyads of mothers and children with NH were recruited from early education centers in the north of Italy. Fathers were not recruited, because most of them claimed to be unavailable for the play sessions prior to the start of the study. All participants spoke – or were in the process of acquiring – Italian only.

The 20 children in the G-NHA group (10 males and 10 females) were individually matched with the G-CI children according to their chronological age at T1 (mean age in the G-CI group = 17.5 months; SD = 8.3; range = 10–35; mean age in the G-NHA group = 17.4; SD = 8.0; and range = 10–34), while the 20 children (9 males and 11 females) in the G-NHL group (mean age = 11.2 months; SD = 0.4; and range = 11–12) were individually matched with the G-CI children according to their linguistic level (lexical production) at T2. Lexical production for matching the children was measured using the “Words and Gestures” of the MacArthur-Bates Communicative Development Inventory (MB-CDI; Italian version by Caselli et al., 2015). *T*-tests showed that the children of the G-CI and G-NHL groups did not differ as regards production scores [*t*(38) = 0.162, *p* = 0.872].

To be enrolled in the study each child with hearing loss had to meet all of the following criteria: (1) diagnosis of severe to profound (>71 dB, following the ASHA classification system; Clark, 1981) bilateral sensorineural hearing loss before 36 months; (2) CI surgery before 36 months; (3) no presence of sensorimotor or developmental disorders; (4) no presence of cognitive disability (IQ > 85); (5) exposure to an oral communication program before and after implantation; (6) normal-hearing parents; and (7) absence of exposure to any other language, including sign language. The last two criteria were chosen to limit within-group variability due to the effect of exposure to another language (for studies on sign language exposure, see for example Davidson et al., 2014; Geers et al., 2017). Children with normal hearing were included in the study if they met the following requirements: bilateral normal hearing, absence of neurodevelopmental disorders (e.g., autism spectrum disorder, neurological pathologies), exposure to Italian only.

Demographic information on the participants and their parents was collected using the background information form included in the MB-CDI test. All the children and mothers tested were Italian monolingual speakers and were of Italian nationality. The Italian MB-CDI background information form does not include questions about the participants’ belonging to specific racial or ethnic groups; therefore, this information was not collected. Maternal education (to use as proxy for socio-economic status) was significantly different between groups, as revealed by an ANOVA [*F*(2, 54) = 4.355, *p* = 0.018]. *Post-hoc* tests with the Tukey correction only showed a significant difference between the G-CI and G-NHL groups, with mean maternal education being significantly lower in the former than in the latter (*t* = −2.943, *p* = 0.013). See Table 1 for mother and child participants’ characteristics.

**TABLE 1:** Participants’ characteristics in the three groups.

	G-CI		G-NHA		G-NHL	
	M	SD	M	SD	M	SD
Age at diagnosis (months)	7.8	9.18				
PTA (dB/HL)	101.25	5.59	/	/	/	/
Age at T1	17.47	8.33	17.42	7.97	11.24	0.42
Age at T2	22.30	8.27	21.91	8.39	15.25	0.31
Age at T3	25.32	8.37	25.67	8.84	19.27	2.24
Age at T4	30.89	8.17	32.02	9.37	24.91	2.71
Vocabulary size at T2	5.40	8.64	34.75	37.71	5.85	6.58
Maternal education (years of study)	13.90	5.10	15.40	3.23	17.70	2.93

**Note*. T1 = first session, for children with hearing loss the day before CI surgery; T2 = second session, for children with hearing loss 3 months after CI activation; T3 = third session, for children with hearing loss 6 months after CI activation; and T4 = fourth session, for children with hearing loss 12 months after CI activation. PTA, Pure Tone Average (250-500-1000-2000-4000 Hz); expressive vocabulary size was measured using the Italian version of the MB-CDI (Caselli et al., 2015).*

Each family signed an informed consent form, and the data were treated in accordance with the current personal data protection code (Legislative Decree no. 196/2003). The protocol for the study was approved by the institutional local ethical committee (protocol number 2017/46268).

### Procedure

Twenty min of each child’s spontaneous language production were observed in the laboratory and video-recorded during mother-child semi-structured play. The observations were conducted using the ALB protocol (Assessing Linguistic Behavior; Olswang et al., 1987). Two different sets of toys were used to elicit spontaneous language production and to keep a high level of engagement, differentiated according to the children’s ages and phases of development. Those given to the children under 15 months were sensory toys, stuffed animals, soft plastic machines and tractors, and soft building blocks; those for children over 15 months were food toys, plastic fruits and vegetables, dishes and cutlery, farm toys, toy cars and tractors, “nurturing” toys, and dolls with beds.

Interactions were video recorded for approximately 20 min, adapting the ALB protocol. Recorded interactions were preceded by a few minutes of warm-up activities to make mother and child feel comfortable. Despite this, one G-CI child from the initial cohort cried during the recordings and had to be excluded from the final sample (*n* = 20). To elicit children’s language production, mothers were instructed to play with their children as they usually do at home, using all toys. All observations were recorded using the same video camera with a built-in 5.1-channel surround microphone. The researcher verified the quality of the recordings after each session.

### Coding

All video-observations were transcribed and codified using CHILDES (the Child’s Language Data Exchange System: MacWhinney, 2000). Utterances judged to be unintelligible after four listenings were excluded. Specifically, babbling production was considered adapting the categories developed by Paul and Jennings (1992) and including production with one consonant or a repetition of the same consonant type (e.g., [da], [tata]) and production with two or more different consonants (e.g., [tadi]).

Verbal language production was also considered. Specifically, tokens (the total number of words) and types (the number of different words) were considered (Majorano and Lavelli, 2014; Majorano et al., 2018). In line with other studies, the number of tokens was regarded as an index of speech amount, while the number of types was assumed to be an index of the variability of the language. Two experts transcribed and coded the video-observations; tokens and types were automatically calculated using CLAN (MacWhinney, 2000) and then checked by the two experts. The inter-rater agreement (Cohen’s k) based on the 20% of the material was 0.86 for transcriptions and 0.84 for coding.

## Results

Children’s preverbal and lexical production per minute were considered for the three groups. In order to test whether our outcome variables were representative of the child’s language development, Spearman correlations were run between the MB-CDI production scores and our tokens and types measures. Results showed that the two measures were moderately correlated with the MB-CDI scores at T1 [tokens: *r*_(s)_ = 0.47, *p* < 0.001; types: *r*_(s)_ = 0.45, *p* < 0.001] and strongly correlated at T2 [tokens: *r*_(s)_ = 0.84, *p* < 0.001; types: *r*_(s)_ = 0.83, *p* < 0.001], at T3 [tokens: *r*_(s)_ = 0.79, *p* < 0.001; types: *r*_(s)_ = 0.80, *p* < 0.001], and at T4 [tokens: *r*_(s)_ = 0.67, *p* < 0.001; types: *r*_(s)_ = 0.74, *p* < 0.001]. Preliminary analysis also showed that the level of tokens and types produced at T4 was not related to the children’s age at the end of the study [tokens: *r*_(s)_ = 0.14, *p* = 0.270; types: *r*_(s)_ = 0.15, *p* = 0.266].

In order to investigate possible differences between groups and sessions in preverbal production, a series of Linear Mixed-Effects Models (LMMs) with Group (with three levels) and Session (with four levels) as fixed effects, Maternal education as covariate, and Subject as random effect were run in R (R Core Team, 2018; “lmer” function in the “lme4” package, Bates et al., 2015). Pairwise comparisons after significant interactions were run with the Holm correction (“testInteractions” function in the “phia” package; De Rosario-Martinez, 2015). The same was carried out for tokens and types; in the latter case, pairwise comparisons after main effects were run with the Tukey correction (“emmeans” function in the “emmeans” package; Lenth et al., 2020).

As regards preverbal production, results showed a significant main effect of Session [*F*(3,162) = 5.227, *p* = 0.002] and a significant Group × Session interaction [*F*(6,162) = 5.628, *p* < 0.001]. *Post-hoc* analysis revealed a significant difference between the G-CI and the G-NHL groups at T4, with the G-CI group producing a significantly higher proportion of babbling 12 months after CI activation [χ^2^_(1)_ = 14.844, *p* = 0.001]. No other significant differences were found between groups (all *p*s > 0.05). Furthermore, *post-hoc* tests indicated that the G-CI group was the only one to have significantly increased proportions of babbling at T3 and T4 as compared to T1 and T2 [T1 vs. T3: χ^2^_(1)_ = 12.492, *p* = 0.006; T1 vs. T4: χ^2^_(1)_ = 27.322, *p* < 0.001; T2 vs. T3: χ^2^_(1)_ = 10.214, *p* = 0.021; T2 vs. T4: χ^2^_(1)_ = 23.897, *p* < 0.001], while the other two groups maintained more similar proportions throughout the four sessions (all *p*s > 0.05).

Data analysis on tokens production showed significant main effects of Group [*F*(2, 53) = 12.817, *p* < 0.001] and Session [*F*(3, 162) = 85.374, *p* < 0.001], and a significant Group and Session interaction [*F*(6, 162) = 2.903, *p* = 0.010]. *Post-hoc* tests showed significant differences between the G-CI and G-NHA groups at T2 [χ^2^_(1)_ = 9.774; *p* = 0.016], T3 [χ^2^_(1)_ = 14.762, *p* = 0.001], and T4 [χ^2^_(1)_ = 27.324, *p* < 0.001], with the G-NHA group producing a significantly higher proportion of tokens. No difference between these groups was found at T1 (*p* = 0.234), possibly because of not enough variance in the number of tokens produced per minute by the children (mean at T1 = 0.69, SD = 1.95). *Post-hoc* tests also showed significant differences between the two normally hearing groups at T1 [χ^2^_(1)_ = 8.822, *p* = 0.021], T2 [χ^2^_(1)_ = 14.091, *p* = 0.002], T3 [χ^2^_(1)_ = 9.494, *p* = 0.016], and T4 [χ^2^_(1)_ = 7.772, *p* = 0.032], with a higher production of tokens for the G-NHA group. *Post-hoc* tests also showed significantly higher proportions at T4 than at any of the previous sessions for the G-CI group [T1 vs. T4: χ^2^_(1)_ = 32.781, *p* < 0.001; T2 vs. T4: χ^2^_(1)_ = 23.951, *p* < 0.001; and T3 vs. T4: χ^2^_(1)_ = 13.500, *p* = 0.002], and significantly higher proportions at T3 as compared to T1 [χ^2^_(1)_ = 22.701, *p* < 0.001] and at T4 as compared to T1 [χ^2^_(1)_ = 107.444, *p* < 0.001], T2 [χ^2^_(1)_ = 61.097, *p* < 0.001], and T3 [χ^2^_(1)_ = 31.371, *p* < 0.001] for the G-NHA group. Finally, the G-NHL groups showed significant differences between T1 and T3 [χ^2^_(1)_ = 18.001, *p* < 0.001], between T1 and T4 [χ^2^_(1)_ = 96.094, *p* < 0.001], between T2 and T3 [χ^2^_(1)_ = 8.707, *p* = 0.022], between T2 and T4 [χ^2^_(1)_ = 72.433, *p* < 0.001], and between T3 and T4 [χ^2^_(1)_ = 30.914, *p* < 0.001].

**FIGURE 1:**
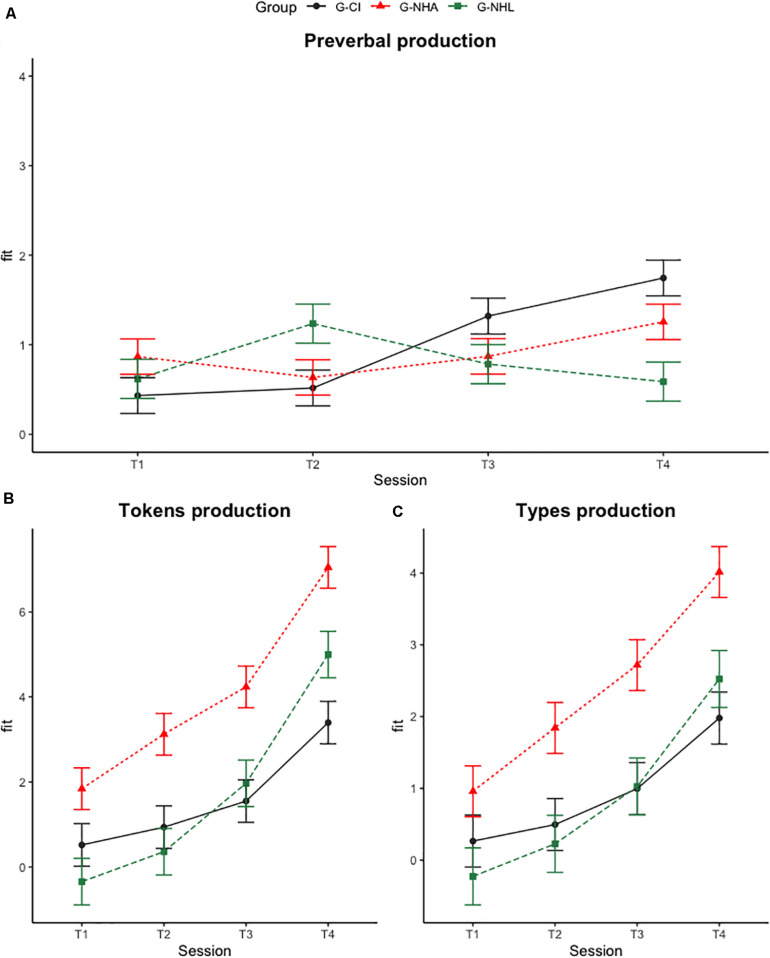
Adjusted group and session means and standard error estimates of the fitted models on babbling **(A)**, tokens **(B)**, and types **(C)**. G-CI: black solid line; G-NHA: red dotted line; and G-NHL: green dashed line.

As for types, data analysis showed again main effects of Group [*F*(2, 53) = 7.964, *p* = 0.001] and of Session [*F*(3, 162) = 66.854, *p* < 0.001]. *Post-hoc* tests with the Tukey correction showed significantly lower proportions of types for the G-CI group (*t* = −3.407, *p* = 0.004) and for the G-NHL group (*t* = −3.320, *p* = 0.005) than for the G-NHA group. Finally, significantly higher production was found at the later sessions as compared to the previous ones (T1 vs. T2: *t* = 2.779, *p* = 0.031; T1 vs. T3: *t* = −6.638, *p* < 0.001; T1 vs. T4: *t* = −13.341, *p* < 0.001; T2 vs. T3; *t* = −3.859, *p* < 0.001; T2 vs. T4: *t* = −10.562, *p* < 0.001; and T3 vs. T4: *t* = −6.703, *p* < 0.001). See Figure 1.

To investigate whether age at diagnosis was associated with the level of lexical development reached at T4 by our CI group, we ran Spearman correlations between age at diagnosis and tokens and types at T4. These showed that both tokens [*r*_(s)_ = −0.46, *p* = 0.044] and types [*r*_(s)_ = −0.58, *p* = 0.007] were inversely correlated with age of diagnosis.

### The Study of Trajectories Using Latent Class Growth Analysis

We conducted latent class growth analysis (LCGA) as a means of identifying growth latent trajectories of preverbal production, tokens, and types, over four time points, using maximum-likelihood estimation to estimate class parameters (Muthén, 2004; Muthén and Muthén, 2006). Then, we used preverbal production at T1 as predictor of the trajectories and of the membership in the classes identified for tokens and types. LCGA was performed in Mplus using the guidelines of Jung and Wickrama (2008). The analysis was conducted in three steps: first, we identified the best baseline model for each outcome measure; then, we explored the latent class growth models without covariates (unconditional models); finally, we analyzed the model including preverbal production at T1 as covariate (conditional model).

#### Identification of the Best Baseline Model

A single class latent growth curve model was run to define the best baseline model for each outcome measure. Two growth curves were compared: a first curve with the measures repeated on the four time points as indicators and with the intercept and linear slope as higher order latent factors, and a second one adding a quadratic parameter. As shown in Table 2, for all measures the model including the quadratic factors had better fit indices (a higher Comparative Fit Index, a lower Root Mean Square Error of Approximation, and a lower Standardized Root Mean Square Residual) and a lower Bayesian Information Criterion (BIC) than the model with the intercept and slope, which indicates that the model including the quadratic parameter provided a better fit to the data. The estimates of variance related to the intercept and slope were significant for all measures: this suggests the presence of more than one trajectory with different growth trends, which justifies an examination of interindividual differences across measures over time; the variance of the quadratic factor was only statistically significant for tokens and types.

**TABLE 2:** Fit indexes, means, and variances of the parameters for the growth models (GM).

Model	χ^2^ (df)	CFI	RMSEA	SRMR	BIC	Mean			Variance		
						Intercept	Slope	Quadratic	Intercept	Slope	Quadratic
**Preverbal production**											
Model with intercept and slope	45.909 (6)	0.83	0.33	0.25	2119.58	8.40**	8.68***	/	550.35***	57.50***	/
Model with intercept, slope, and quadratic parameter	11.047 (1)	0.96	0.41	0.05	2105.19	8.16***	1.75	4.10***	578.44**	96.63	8.71
**Tokens**											
Model with intercept and slope	50.835 (6)	0.76	0.35	0.20	1008.31	0.69***	1.06***	/	3.75***	0.65***	/
Model with intercept, slope, and quadratic parameter	11.466 (3)	0.95	0.22	0.08	981.22	0.69***	0.34	0.39***	3.75***	1.56**	0.30***
**Types**											
Model with intercept and slope	68.695 (6)	0.76	0.42	0.21	758.53	0.35*	0.67***	/	1.25***	0.24***	/
Model with intercept, slope, and quadratic parameter	6.089 (2)	0.98	0.19	0.06	712.30	0.35*	0.33*	0.17**	1.25***	0.94***	0.15**

*Note. N* = 108. *df*, Degrees of freedom; CFI, Comparative Fit Index (higher values indicate a better fit); RMSEA, Root Mean Square Error of Approximation (lower values indicate a better fit); SRMR, Standardized Root Mean Square Residual (lower values indicate a better fit); and BIC, Bayesian Information Criterion (lower values indicate a better fit).* *p* < 0.05, ** *p* < 0.01, and *** *p* < 0.001.

#### Exploration of the Latent Class Growth Models Without Covariates (Unconditional Models)

As a second step, we specified a latent class growth model without covariates (unconditional). We assessed the best fitting model on the basis of the number of latent classes and of the best fitting parameters (linear vs. linear and quadratic). In order to choose the best models, we considered the fit indices and information criteria. We used the BIC and the Bootstrap Likelihood Ratio Test (B-LRT). In addition, we followed the recommendations from the literature (e.g., Nylund et al., 2007), considering interpretability and parsimony as discriminating criteria. We also evaluated entropy values, to assess the degree of separation among the classes in the models, where scores closer to 1 highlight better fit of the data; the proportions for the latent classes (not less than 1% of total count in a class); and the posterior latent class probabilities (near to 1.00). For all the measures we compared progressive unconditional models from one to four classes examining them on the basis of different elements.

For preverbal production the best solution was a model with intercept and slope factors and three classes. As shown in Table 3, the BIC was lower in the linear and quadratic models with three classes than in the models with two classes, and lowest in the linear model with three classes; despite the entropy of the linear model with three classes was lower than that of the model including the quadratic factor, the former had more homogeneous latent class proportions.

**TABLE 3:** Information criteria and fit indexes for the unconditional LCGM models.

	Linear unconditional model (no covariates)						
	Number of Classes	Parameters*	BIC	B-LRT *p*-value	Entropy	Number of subjects (%) in each class	Posterior probability estimate of class membership
Preverbal	Linear C2	9	630.80	<0.001	0.89	13 (21%), 47 (79%)	0.94, 0.97
	Quadratic C2	11	637.30	<0.001	0.91	10 (17%), 50 (83%)	0.89, 0.99
	Linear C3	12	625.62	<0.001	0.87	41 (68%), 13 (22%), 6 (10%)	0.96, 0.92, 0.88
	Quadratic C3	15	629.90	<0.001	0.91	44 (73%), 12 (20%), 4 (7%)	0.97, 0.94, 0.87
Tokens	Linear C2	9	980.23	<0.001	1.00	4 (7%), 56 (93%)	1.00, 1.00
	Quadratic C2	11	974.03	<0.001	1.00	56 (93%), 4 (7%)	1.00, 1.00
	Linear C3	13	927.78	<0.001	0.92	10 (17%), 4 (7%), 46 (76%)	0.90, 1.00, 0.97
	Quadratic C3	15	930.45	<0.001	0.97	47 (78%), 9 (15%), 4 (7%)	0.99, 0.96, 1.00
Types	Linear C2	9	722.06	<0.001	1.00	56 (93%), 4 (7%)	1.00, 1.00
	Quadratic C2	11	696.62	<0.001	1.00	56 (93%), 4 (7%)	1.00, 1.00
	Linear C3	12	662.37	<0.001	1.00	56 (93%), 1 (2%), 3 (5%)	1.00, 1.00, 1.00
	Quadratic C3	15	655.24	<0.001	1.00	3 (5%), 56 (93%), 1 (2%)	1.00, 1.00, 1.00

*Note.* BIC, Bayesian Information Criterion; B-LRT, Bootstrap Likelihood Ratio Test. C2, two classes; C3, three classes.* Intercept and slope variance fixed to zero in some cases.

**FIGURE 2:**
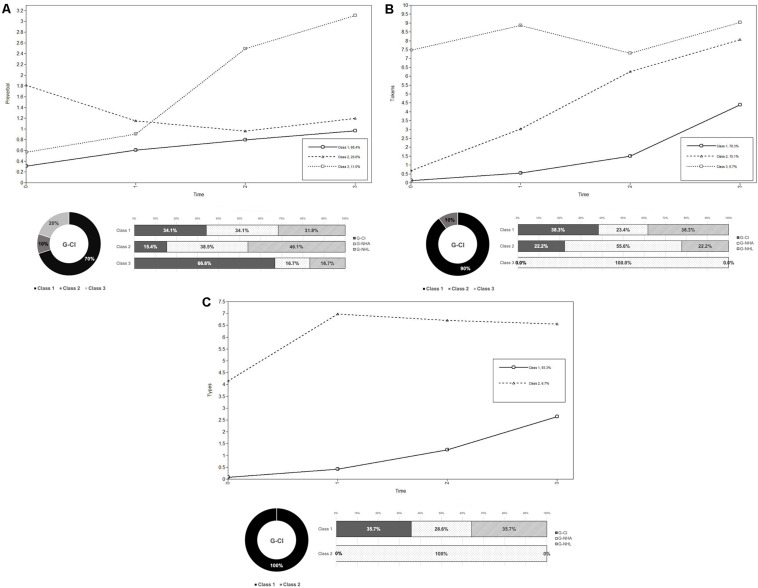
Babbling **(A)**, tokens **(B)**, and types **(C)** by class and session. Each panel shows (i) the trajectories of the classes identified by the unconditional models (observed means; class 1: solid line; class 2: dashed line; class 3: dotted line), (ii) the distribution of the G-CI children across classes (bottom left), and (iii) the composition of each class (bottom right). *Note*. Preverbal class distributions: 14 G-CI children, 14, G-NHA children, and 13 G-NHL children in class 1; two G-CI children, five G-NHA children, and six G-NHL children in class 2; four G-CI children, one G-NHA child, and one G-NHL child in class 3. Tokens class distributions: 18 G-CI children, 11 G-NHA children, and 18 G-NHL children in class 1; two G-CI children, five G-NHA children, and two G-NHL children in class 2; four G-NHA children in class 3. Types class distributions: 20 G-CI children, 16 G-NHA children, 20 G-NHL children in class 1; four G-NHA children in class 2.

The selected model for preverbal production had three classes, that is, three clusters of individuals that follow the same developmental pattern over time (see Figure 2). Class 1 had an increasing curve and involved 68% of our participants (*n* = 41): of these, 14 belonged to the G-CI group, 14 belonged to the G- NHA group, and 13 belonged to the G- NHL group. Class 2 had a decreasing trend and was composed of 13 participants (21% of the sample): two from the G-CI group, five from the G-NHA group, and six from the G-NHL group. Class 3, constituted by six participants (11%; four children from the G-CI group, one child from the G-NHA group, and one child from the G-NHL group) had a steeper increasing curve (see Figure 2).

The analysis for tokens revealed that the best solution was a model with intercept, slope and quadratic factors and three classes (see Table 3). Even though the four-class model was the best according to the B-LRT (*p* < 0.001) and to the fact that its BIC was slightly lower (875.65), its latent class proportions were inadequate; for these reasons, the four-class model was rejected in favor of the three-class model. The exploration of the trajectories confirmed the goodness of the three-class model, showing two trajectories with an increasing trend and one trajectory with a more stable pattern of tokens production over time (see Figure 2). Class 1 (showing an increasing trend) was constituted by 18 children from the G-CI group, 11 children from the G- NHA group, and 18 children from the G-NHL group. Class 2 (with a steeper increasing curve) included two children from the G-CI group, five children from the G-NHA group, and two children from the G-NHL group. Only four children (all from the G-NHA group) had a more stable trajectory and were in class 3.

Analysis showed that the best model to describe the trajectories for types was the one with linear and quadratic factors and two latent classes. In fact, even though the model with a quadratic factor and three classes showed a slightly lower BIC, the distribution of the participants in the classes and the parsimony criterion suggested that the model to be preferred was the one with two latent classes (see Table 3). However, this solution seemed not to adequately discriminate differences in the trajectories over time: class 1 was constituted by 93% of our sample and showed a slowly increasing curve: in this class there were 20 children from the G-CI group, 16 children from the G-NHA, and 20 children from the G-NHL group; class 2 (with a curve that was steep between T1 and T2 and then almost flat) was constituted by only four children from the G-NHA group, and one child from the G-NHL group) had a steeper increasing curve (see Figure 2).

#### Analysis of the Conditional Model

Next, we added into our (previously unconditional) models on tokens and types preverbal production at time 1 as covariate (thus creating a conditional model); due to sample size, it was not possible to add maternal education as additional covariate. Table 4 shows the parameters’ estimates for the unconditional and conditional models for tokens.

**TABLE 4:** Parameters’ estimates, information criteria, and fit indexes for the unconditional and conditional models.

	Trajectories of tokens over time					
	Unconditional LCGM			Conditional LCGM^1^		
	Slower increasing	Steeper increasing	Constant high	Constant high	Slower increasing	Steeper increasing
Mean intercept	0.14**	0.65°	7.54***	7.54***	0.07	0.25
Mean slope	−0.22°	2.55***	1.13	1.06	−0.27	0.55
Mean quadratic	0.51***	0.04	−0.38	−0.25	0.35*	0.53
Intercept on preverbal	−	−	−	−1.83***	−	−
Slope on preverbal	−	−	−	−	−	−
Quadratic on preverbal	−	−	−	−	0.18*	−
Number of subjects (%) in each class	47 (78)	9 (15)	4 (7)	5 (8)	40 (67)	15 (25)
Posterior probability of class membership	0.99	0.96	1.00	1.00	0.97	0.93
**Estimated parameters**	15			32		
BIC	930.45			916.99		
B-LRT *p*-value	<0.001			<0.001		
Entropy	0.97			0.92		

*Note.* BIC, Bayesian Information Criterion; B-LRT, Bootstrap Likelihood Ratio Test.^1^The conditional model included preverbal production at T1 as covariate. Its effect was estimated only when it was statistically significant.*p < 0.05, **p < 0.01, ***p < 0.001, and °*p* < 0.10.

**FIGURE 3:**
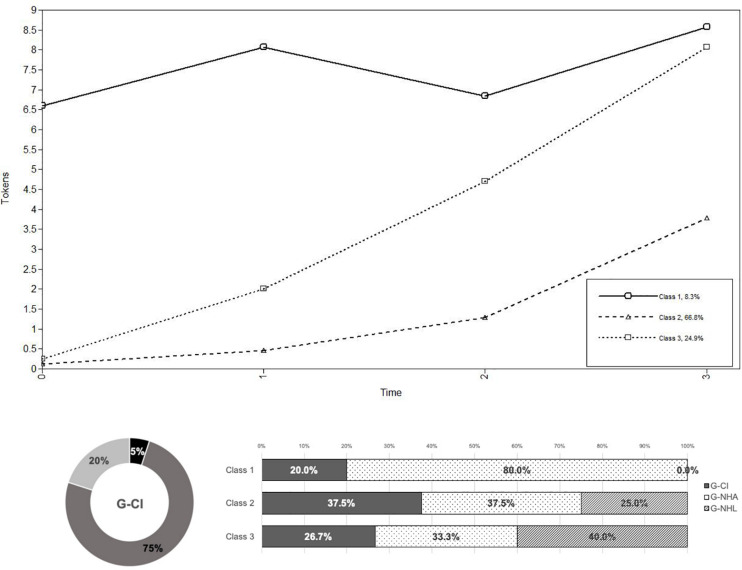
The three trajectories (class 1: solid line; class 2: dashed line; class 3: dotted line) of tokens production over time, as identified by the model including preverbal production at T1 as covariate (observed means). The plot on the bottom left shows the distribution of the G-CI children across classes; the plot on the bottom right shows the composition of each class. *Note.* Tokens class distribution: one G-CI child, four G-NHA children in class 1; 15 G-CI children, 15 G-NHA children, and 10 G-NHL children in class 2; four G-CI children, five G-NHA children, and six G-NHL children in class 3.

The best conditional model for tokens had linear and quadratic factors and three latent classes, thus confirming the solution found while analyzing the unconditional model. Five participants (8% of our total sample) showed a high and stable production of tokens over time (class 1; “Constant high”): of these, one child was from the G-CI group, while the other four children were from the G-NHA group; forty children (67%) showed a slower increasing curve (class 2; “Slower increasing”): of these, 15 children were from the G-CI group, 15 children were from the G-NHA group, and 10 children were from the G-NHL group; finally, the remaining fifteen children (25%) showed a steep curve with rapid increases in tokens production over time (class 3; “Steeper increasing”): of these, four children were from the G-CI group, five children from the G-NHA group, and six children from the G-NHL group (see Figure 3).

Results showed that preverbal production had a negative effect on the intercept for the Constant High tokens class and a positive effect on the quadratic factor for the Slower Increasing tokens class; no effect was found for the Steeper Increasing tokens class. The effect of preverbal production on class membership was studied by designating the Slower Increasing tokens class (comprising the largest number of participants: *n* = 40) as the reference class, and by using logistic regressions to assess the degree to which the probability of being in that class was associated with the covariate. Tests showed no statistically significant effect of preverbal production on class membership. In short, the level of preverbal production at T1 did not affect class membership and the lexical growth profile of the children; however, how much children babbled at T1 had different effects on tokens production across classes: for the children with a more stable trajectory of lexical development, initial lower preverbal production was associated with higher tokens production; for the children who slowly increased their tokens production over time, higher preverbal production was nonlinearly associated with higher tokens production, especially later in time (see Figure 3).

Based on the BIC, B-LRT, and entropy values, as well as on the posterior probability estimates, the best conditional model on types included a linear and a quadratic factor with three classes (see Table 3). However, this solution resulted unstable concerning class membership and was not retained.

## Discussion

The aim of the study was to assess the preverbal and lexical development of children with CIs before surgery and at three and 6 months after CI activation, and to compare their results with those of two groups of NH children, one chronologically age matched (G-NHA) and one matched for level of lexical production (G-NHA). Furthermore, we aimed at assessing the possible role of babbling in affecting lexical growth in the three groups.

As regards the children’s preverbal trajectories, data showed significant increases in babbling production between sessions for the children with CIs; as we expected, at 12 months after implantation the amount produced by this group was comparable to that of the age-matched group and significantly higher than that of the children with a similar lexical level (G-NHL). This finding is in line with other studies showing a higher increase of babbling in children after CI activation, but similar proportions of babbling in children with CIs and in age-matched peers (Moore and Bass-Ringdahl, 2002; Schramm et al., 2010) and higher proportions in children with CIs as compared to children with similar language skills (Ertmer and Inniger, 2009). The fact that children with CIs significantly increase their production of babbling between 3 and 6 months after implantation, and produce a higher proportion of babbling as compared to children with similar language development at 12 months may indicate two things: first, that the ability to hear now offers these children the possibility to practice their speech motor routines; second, that CI children may continue to babble even after having acquired new words, probably due to their need to establish a stable connection between word auditory processing and phono-articulatory skills. CIs allow children to have feedback on their own production; this may lead them to increase their use of babbling both as articulatory exercise and to have a match between articulatory information and (the newly-established) auditory feedback. This process has the effect to facilitate the formation of stable auditory-motor representations of speech sounds (Bruner, 1964; Thelen, 1981; Lickliter and Bahrick, 2004; Fagan, 2015; see Kuhl et al., 2014 for neuroimaging data supporting this point). In contrast, the absence of increases in babbling production between sessions for the children with the same chronological age (G-NHA) may be due to their increasing lexical productions or the age at which they were tested (the time period with the most robust increases in babbling may have passed). The children matched for linguistic level also displayed stable babbling production over time; their lower proportions as compared to the G-CI group may be due to their less advanced physical, cognitive, and social maturity (Ertmer and Inniger, 2009).

In line with our hypotheses regarding the children’s lexical trajectories, results showed that all participants displayed a significant increase in word production over time; however, the children with CIs produced a significantly lower number of tokens as compared to the G-NHA children at any session after CI activation, and a comparable number of tokens as the G-NHL group at 12 months after CI activation. Interestingly, preliminary analysis excluding maternal education as covariate had shown a significant difference between the G-CI and G-NHL groups at T4, with the former producing fewer tokens. The finding that differences in word production levels disappear when maternal education is accounted for confirms previous research studies showing significant effects of socio-economic status and maternal education on child language (Hoff, 2006) and suggests that delays in lexical production may be worsened by an unfavorable home linguistic environment (Szagun and Schramm, 2016). As for types, we only found main group and session differences; more specifically, lower lexical diversity was found in the G-CI and G-NHL groups as compared to the G-NHA group, and at each session as compared to all of the previous ones. The fact that types production is lower for CI children than for their age-matched peers, but not as compared to their language-matched peers, suggests a lexical delay for the G-CI group. Nonetheless, results indicated significant improvements between sessions in types production in general; moreover, although we did not find significant group and session interactions, Figure 1 seems to show a similar – but slower – positive trend for the G-CI group, which suggests that children with CIs may eventually catch-up with their peers a few months later, as shown in other studies relative to vocabulary size (Miyamoto et al., 1997; Kirk et al., 2002; Svirsky et al., 2004; Tomblin et al., 2005; Duchesne et al., 2009), possibly thanks to their early CI activation (before 36 months of age). In line with this idea, we found that earlier diagnosis was associated with a higher level of tokens and types produced at T4, and thus with a higher level of lexical development one year after CI activation. In sum, the G-NHA and G-NHL children presented a level of language development that is in line with their chronological age (the second half of the first year; Menyuk et al., 1986; Whitehurst et al., 1991; Ferguson et al., 1992; Vihman, 1996, 2014; Kuhl et al., 2008), while the children with CIs showed linguistic (Oller et al., 1999; Kishon-Rabin et al., 2005).

As reported in previous studies, at around 14–19 months (corresponding to T1 for the G-NHA and G-CI children and to T2 for the G-NHL group) children tend to extend their lexical and phonological skills by increasing their vocabulary size and by using more complex phonological patters, usually exercised in preverbal utterances. The lexical delay of the G-CI children may depend on their more limited practice with matching self-produced syllables with patterns perceived in the input (Keren-Portnoy et al., 2009; Majorano et al., 2014). Only after CI activation would children with hearing loss be able to considerably increase their sounds production and phonetic inventory in preverbal and lexical production, and, as a consequence, extend their vocabulary (Schauwers et al., 2004; Walker and Bass-Ringdahl, 2008; Schramm et al., 2010; McDaniel and Gifford, 2020). To investigate the relationship between preverbal skills and lexical development in the three groups, we analyzed the children’s growth latent trajectories of preverbal production, as well as of tokens and types, over four time points, while taking into account the effect of preverbal production. First, it should be noted that preliminary latent growth analysis of the trajectories for babbling and tokens revealed different trajectories within the CI group. As a matter of fact, tests showed that, despite most children had an increasing curve for babbling, four of them showed larger increases between sessions as compared to the other peers (especially after implantation), while two others decreased their preverbal production over time, possibly because they were in the process of expanding their lexical repertoire in the meantime. Different within-group trajectories were also found with respect to tokens production: all children showed increasing word production over time, but two children showed a steeper growth trajectory. Interestingly, these two children had a similar profile, as they had both been diagnosed at 2 months of age and implanted at 13 months (earlier with respect to the group mean, see Table 1) and had mothers with a higher level of education (mean = 21 years; group mean = 13.9). Moreover, in line with the idea that lower levels of babbling production may also indicate a more advanced lexical development, one of the two children in this tokens class belonged to the class with a decreasing babbling curve; the other child in the decreasing babbling class also showed a positive tokens trend. These results are in line with previous studies showing that children with CIs display great variability in their babbling and lexical developmental profiles after CI activation (McDaniel and Gifford, 2020). Moreover, comparison with the other groups shows similar tokens class distributions for the children with CIs and their language-matched peers, with eighteen children showing an increasing curve, two children showing an even steeper curve, and no child showing a decreasing or stable trend within each group. It should also be noted that the class with the steeper growth curve is constituted by more children from the age-matched group than from the other two groups, which suggests that more rapid and significant advances in lexical production are more common for the normal hearing group over the second year of life. The present findings suggest that within the first year after CI activation most children with hearing loss show preverbal characteristics in their vocal productions, probably because their basic phonetic inventory and phonological skills are still fragile and cannot support a more advanced vocabulary expansion.

As regards the role of babbling in supporting vocabulary production, our data showed differences relating to the children’s lexical trajectories. In fact, while babbling production at T1 did not have a statistically significant effect on class membership, it did have effects on tokens production for groups with slower increasing and stable trajectories. Interestingly, these effects were opposite in the two groups: higher levels of babbling production at T1 (which signal a lower level of early lexical development) were associated with lower tokens production in children with a high and stable lexical production trajectory (mostly, the age-matched NH children), while they seemed to support lexical advancements in children who were developing their language skills more slowly. Given that most of the children with CIs are in the “slower” group, it can be concluded that babbling production has a significant role in supporting the lexical development of children with hearing loss. As reported in previous studies, a delay in canonical babbling is associated with subsequent fragility in language areas such as lexical production (Oller and Eilers, 1988; Stoel-Gammon, 1988), phonological articulation (Moeller et al., 2007), and literacy acquisition (Lieu, 2013). This delay, and the lost opportunities to practice their early phono-articulatory skills as well as to create sensorimotor representations connecting environmental input and output, may be even more detrimental for children with hearing loss.

It should be pointed out that the sample included in the present study may not be large enough for subtle differences across children to appear. Despite being larger than that of the majority of the previous studies in the field (McDaniel and Gifford, 2020), our sample size did not allow the addition of multiple covariates to our models to account for effects of factors such as maternal education, and only allowed the use of latent class growth models, which – despite providing a clear identification of classes without a high computational burden – assume no within-class variance on the growth factors. More refined tests, such as growth mixture models, may be of great benefit to the analysis of children’s lexical trajectories, especially for categories such as types. Matching the groups on socio-economic status prior to the start of the study may also partly solve the issue. Moreover, despite good correspondence between vocabulary scores and tokens/types measures in our data, the 20-min time limit of the recordings may not provide a comprehensive assessment of the children’s language abilities. Longer recordings, possibly collected using the Language Environment Analysis (LENA) software, may increase the reliability of our results. Of interest for future studies would also be the analysis of father-child interactions (which are just as important for children’s development, see Volling et al., 2019) and the addition of an observation at 24 months, so as to investigate whether children with CIs have caught up with their peers by 2 years after CI activation; future research should also ideally aim to include a narrower age range to obtain a clearer picture of how language is developed in this population.

Nonetheless, the study provides important empirical support in favor of the connection between babbling and early lexical development, showing both common patterns and individual differences in the CI children’s developmental trajectories. The results relating to the relationship between preverbal and lexical development, in particular, offer insights into the language development process of children with CIs, and may have important implications for future clinical observational assessment and language rehabilitation programs before and after CI activation. Our findings suggest that professionals and families should focus more attention on preverbal speech characteristics; if the link between babbling and later verbal production were corroborated by substantial experimental findings in the future, preverbal production might become an important risk factor for the early identification of language delay in children with CIs.

## Data Availability Statement

The raw data supporting the conclusions of this article will be made available by the authors, without undue reservation.

## Ethics Statement

The studies involving human participants were reviewed and approved by the Piacenza hospital ethical committee (protocol number 2017/46268). Written informed consent to participate in this study was provided by the participants’ legal guardian/next of kin.

## Author Contributions

MMa: conceptualization, writing (lead), and editing. MB and VP: statistical analysis, writing, and editing. MMo: data collection. RF: data collection and writing. ML: conceptualization and editing. LG: data collection and conceptualization. DC: conceptualization.

## Conflict of Interest

The authors declare that the research was conducted in the absence of any commercial or financial relationships that could be construed as a potential conflict of interest.

## References

[B1] Apuzzo M. L. Yoshinaga-Itano C. (1995). Early identification of infants with significant hearing loss and the minnesota child development inventory. *Semin. Hear.* 16 124–135. 10.1055/s-0028-1083710

[B2] Bates D. Mächler M. Bolker B. Walker S. (2015). Fitting linear mixed-effects models using lme4. *J. Stat. Softw.* 67 1–48. 10.18637/jss.v067.i01

[B3] Bloom K. Russell A. Wassenberg K. (1987). Turn taking affects the quality of infant vocalizations. *J. Child Lang.* 14 211–227. 10.1017/S0305000900012897 3611239

[B4] Bruner J. S. (1964). The course of cognitive growth. *Am. Psychol.* 19 1–15. 10.1037/h0044160

[B5] Caselli M. C. Bello A. Rinaldi P. Stefanini S. Pasqualetti P. (2015). *Il Primo Vocabolario Del Bambino: Gesti, Parole e Frasi. Valori di riferimento fra 8 e 36 mesi delle forme complete e delle forme brevi del questionario MacArthur-Bates CDI.* Milano: Franco Angeli.

[B6] Clark J. G. (1981). Uses and abuses of hearing loss classification. *ASHA* 23 493–500. 7052898

[B7] Davidson K. Lillo-Martin D. Chen Pichler D. (2014). Spoken English language development among native signing children with cochlear implants. *J. Deaf Stud. Deaf Educ.* 19 238–250. 10.1093/deafed/ent045 24150489 PMC3952677

[B8] Davis B. L. MacNeilage P. F. (2000). On the origin of the internal structure of words forms. *Science* 288 527–531. 10.1126/science.288.5465.527 10775113

[B9] De Rosario-Martinez H. (2015). *‘phia: Post-Hoc Interaction Analysis. R package version 0.2-1’.* Available online at: https://cran.r-project.org/package=phia (accessed September 16, 2020).

[B10] DesJardin J. L. Doll E. R. Stika C. K. Eisenberg L. S. Johnson K. J. Ganguly D. H. (2014). Parental support for language development during joint book reading for young children with hearing loss. *Commun. Disord. Q.* 35 167–181. 10.1177/1525740113518062 25309136 PMC4191727

[B11] Dettman J. Pinder J. Briggs C. Dowell R. Leigh R. (2007). Communication development in children who receive the cochlear implant younger than 12 months: risks versus benefits. *Ear Hear.* 28 11S–18S. 10.1097/AUD.0b013e31803153f8 17496638

[B12] Duchesne L. Sutton A. Bergeron F. (2009). Language achievement in children who received cochlear implants between 1 and 2 years of age: group trends and individual patterns. *J. Deaf Stud. Deaf Educ.* 14 465–485. 10.1093/deafed/enp010 19461113

[B13] Ertmer D. J. Inniger K. J. (2009). Characteristics of the transition to spoken words in two young cochlear implant recipients. *J. Speech Lang. Hear. R.* 52 1579–1594. 10.1044/1092-4388(2009/06-0145) PMC2831210 19717658

[B14] Ertmer D. J. Mellon J. A. (2001). Beginning to talk at 20 months. *J. Speech Lang. Hear. R.* 44 192–206. 10.1044/1092-4388(2001/017) 11218103

[B15] Ertmer D. J. Young N. Grohne K. Mellon J. A. Johnson C. Corbett K. (2002). Vocal development in young children with cochlear implants: profiles and implications for intervention. *Lang. Speech Hear. Ser.* 33 184–195. 10.1044/0161-1461(2002/016) 27764399

[B16] Fagan M. K. (2015). Why repetition? Repetitive babbling, auditory feedback, and cochlear implantation. *J. Exp. Child Psychol.* 137 125–136. 10.1016/j.jecp.2015.04.005 25974171 PMC4442053

[B17] Ferguson C. A. Menn L. Stoel-Gammon C. (1992). *Phonological Development: Models, Research, Implication.* Timonium, MD: York Press.

[B18] Fry D. B. (1966). “The development of the phonological system in the normal and the deaf child,” in *The Genesis of Language*, eds Smith F. Miller G. A. (Cambridge, MA: MIT Press), 187–206.

[B19] Geers A. E. (2006). “Spoken language in children with cochlear implants,” in *Perspectives on Deafness. Advances in the Spoken Language Development of Deaf and Hard-Of-Hearing Children*, eds Spencer P. E. Marschark M. (Oxford: Oxford University Press), 244–270.

[B20] Geers A. E. Mitchell C. M. Warner-Czyz A. Wang N.-Y. Eisenberg L. S. the CDaCI Investigative Team (2017). Early sign language exposure and cochlear implantation benefits. *Pediatrics* 140:e20163489. 10.1542/peds.2016-3489 28759398 PMC5495521

[B21] Goldstein M. H. Schwade J. A. (2008). Social feedback to infants’ babbling facilitates rapid phonological learning. *Psychol. Sci.* 19 515–523. 10.1111/j.1467-9280.2008.02117.x 18466414

[B22] Gros-Louis J. West M. J. Goldstein M. H. King A. P. (2006). Mothers provide differential feedback to infants’ prelinguistic sounds. *Int. J. Behav. Dev.* 30 509–516. 10.1177/0165025406071914

[B23] Hoff E. (2006). How social contexts support and shape language development. *Dev. Rev.* 26 55–88. 10.1016/j.dr.2005.11.002

[B24] Hoff E. (2013). *Language Development. Fifth Edition.* Hampshire: Cengage Learning.

[B25] Jung J. Houston D. (2020). The relationship between the onset of canonical syllables and speech perception skills in children with cochlear implants. *J. Speech Lang. Hear. R.* 63 393–404. 10.1044/2019_JSLHR-19-00158 PMC7210441 32073331

[B26] Jung T. Wickrama K. A. S. (2008). An introduction to latent class growth analysis and growth mixture modeling. *Soc. Pers. Psychol. Compass* 2 302–317. 10.1111/j.1751-9004.2007.00054.x

[B27] Keren-Portnoy T. Majorano M. Vihman M. (2009). From phonetics to phonology: the emergence of first words in Italian. *J. Child Lang.* 36 235–267. 10.1017/S0305000908008933 18789180

[B28] Kirk K. I. Miyamoto R. T. Lento C. L. Ying E. O’Neill T. Fears B. (2002). Effects of age at implantation in young children. *Ann. Otol. Rhinol. Laryngol. Suppl.* 111 69–73. 10.1177/00034894021110s515 12018353

[B29] Kishon-Rabin L. Taitelbaum-Swead R. Ezrati-Vinacour R. Hildesheimer M. (2005). Prelexical vocalization in normal hearing and hearing-impaired infants before and after cochlear implantation and its relation to early auditory skills. *Ear Hear.* 26 17S–29S. 10.1097/00003446-200508001-00004 16082264

[B30] Kuhl P. K. Conboy B. T. Coffey-Corina S. Padden D. Rivera-Gaxiola M. Nelson T. (2008). Phonetic learning as a pathway to language: new data and native language magnet theory expanded (NLM-e). *Philos. T. Roy. Soc. B* 363 979–1000. 10.1098/rstb.2007.2154 17846016 PMC2606791

[B31] Kuhl P. K. Meltzoff A. N. (1982). The bimodal perception of speech in infancy. *Science* 218 1138–1141. 10.1126/science.7146899 7146899

[B32] Kuhl P. K. Ramirez R. R. Bosseler A. Lin J. L. Imada T. (2014). Infant’s brain responses to speech suggest analysis by synthesis. *Proc. Natl. Acad. Sci. U.S.A.* 111 11238–11245. 10.1073/pnas.1410963111 25024207 PMC4128155

[B33] Lenth R. V. Buerkner P. Herve M. Love J. Riebl H. Singmann H. (2020). *emmeans: Estimated Marginal Means, aka LeastSquares Means. R package version 1.5.0.* Available online at: https://CRAN.R-project.org/package=emmeans (accessed September 8, 2020).

[B34] Lickliter R. Bahrick L. E. (2004). “Perceptual development and the origins of multisensory responsiveness,” in *Handbook of Multisensory Processes*, eds Calvert G. Spence C. Stein B. E. (Cambridge, MA: MIT Press), 643–654.

[B35] Lieu J. E. (2013). Unilateral hearing loss in children: speech-language and school performance. *B-ENT* 21 107–115. 24383229 PMC4382076

[B36] Lindblom B. (2000). Developmental origins of adult phonology: the interplay between phonetic emergents and evolutionary adaptations. *Phonetica* 57 297–314. 10.1159/000028482 10992149

[B37] Locke J. (2001). A Theory of neurolinguistic development. *Brain Lang.* 58 265–326. 10.1006/brln.1997.1791 9182750

[B38] Löfkvist U. Anmyr L. Henricson C. Karltorp E. (2019). Executive functions, pragmatic skills and mental health in children with congenital cytomegalovirus (CMV) infection with cochlear implants: a pilot study. *Front. Psychol.* 10:2808. 10.3389/fpsyg.2019.02808 31998167 PMC6965306

[B39] MacWhinney B. (2000). The CHILDES project: tools for analysing talk: volume I: transcription format and programs, Volume II: the database. *Comput. Linguist.* 26 657–657. 10.1162/coli.2000.26.4.657 32495221

[B40] Majorano M. Guidotti L. Guerzoni L. Murri A. Morelli M. Cuda D. (2018). Spontaneous language production of Italian children with cochlear implants and their mothers in two interactive contexts. *Int. J. Lang. Comm. Dis.* 53 70–84. 10.1111/1460-6984.12327 28560776

[B41] Majorano M. Lavelli M. (2014). Maternal input to children with specific language impairment during shared book reading: is mothers’ language in tune with their children’s production? *Int. J. Lang. Comm. Dis.* 49 204–214. 10.1111/1460-6984.12062 24224893

[B42] Majorano M. Vihman M. M. DePaolis R. A. (2014). The relationship between infants’ production experience and their processing of speech. *Lang. Learn. Dev.* 10 179–204. 10.1080/15475441.2013.829740

[B43] Mayberry R. I. Squires B. (2006). Sign language: acquisition. *Encyclopedia Lang. Linguist.* 11 739–743.

[B44] McDaniel J. Gifford R. H. (2020). Prelinguistic vocal development in children with cochlear implants: a systematic review. *Ear Hear.* [Epub ahead of print]. 10.1097/AUD.0000000000000829 32053545

[B45] McGillion M. Herbert J. S. Pine J. Vihman M. dePaolis R. Keren-Portnoy T. (2017). What paves the way to conventional language? The predictive value of babble, pointing, and socioeconomic status. *Child Dev.* 88 156–166. 10.1111/cdev.12671 27859008

[B46] Menyuk P. Liebergott J. Schultz M. (1986). “Predicting phonological development,” in *Precursors of Early Speech*, eds Lindblom B. Zetterstrom R. (Basingstoke: The Macmillan Press), 79–94.

[B47] Miyamoto R. T. Svirsky M. A. Robbins A. M. (1997). Enhancement of expressive language in prelingually deaf children with cochlear implants. *Acta Oto Laryngol.* 117 154–157. 10.3109/00016489709117758 9105437

[B48] Moeller M. P. Hoover B. Putman C. Arbataitis K. Bohnenkamp G. Peterson B. (2007). Vocalizations of infants with hearing loss compared with infants with normal hearing: part 1-phonetic development. *Ear Hear.* 28 605–627. 17804976 10.1097/AUD.0b013e31812564ab

[B49] Moore J. A. Bass-Ringdahl S. (2002). Role of infant vocal development in candidacy for and efficacy of cochlear implantation. *Ann. Oto. Rhinol. Laryn.* 111 52–55. 10.1177/00034894021110S511 12018349

[B50] Muthén B. (2004). “Latent variable analysis: growth mixture modeling and related techniques for longitudinal data,” in *Handbook of Quantitative Methodology for the Social Sciences*, ed. Kaplan D. (Newbury Park, CA: Sage Publications), 345–368.

[B51] Muthén L. K. Muthén B. (2006). *Mplus User’s Guide*, 4th Edn Los Angeles, CA: Muthén and Muthén.

[B52] Nicholas J. G. Geers A. E. (2007). Will they catch up? The role of age at cochlear implantation in the spoken language development of children with severe to profound hearing loss. *J. Speech Lang. Hear. R.* 50 1048–1062. 10.1044/1092-4388(2007/073) PMC2882067 17675604

[B53] Nott M. Cowan M. Brown M. Wigglesworth M. (2009a). Early language development in children with profound hearing loss fitted with a device at a young age: part I—The time period taken to acquire first words and first word combinations. *Ear Hear.* 30 526–540. 10.1097/AUD.0b013e3181a9ea14 19739282

[B54] Nott M. Cowan M. Brown M. Wigglesworth M. (2009b). Early language development in children with profound hearing loss fitted with a device at a young age: part II Content of the first lexicon. *Ear Hear.* 30 541–551. 10.1097/AUD.0b013e3181aa00ea 19581807

[B55] Nylund K. L. Asparouhov T. Muthén B. O. (2007). Deciding on the number of classes in latent class analysis and growth mixture modeling: a Monte Carlo simulation study. *Struct. Equc. Model.* 14 535–569. 10.1080/10705510701575396

[B56] Oller D. K. Eilers R. E. (1988). The role of audition in infant babbling. *Child Dev.* 59 441–449. 10.2307/1130323 3359864

[B57] Oller D. K. Eilers R. E. Neal A. R. Schwartz H. K. (1999). Precursors to speech in infancy: the prediction of speech and language disorders. *J. Commun. Disord.* 32 223–245. 10.1016/s0021-9924(99)00013-1 10466095

[B58] Olswang L. Stoel-Gammon C. Coggins T. Carpenter R. (1987). *Assessing Linguistic Behaviour (ALB).* Seattle: University of Washington Press.

[B59] Paul R. Jennings P. (1992). Phonological behaviour in toddlers with slow expressive language development. *J. Speech Hear. R.* 35 99–107. 10.1044/jshr.3501.99 1735981

[B60] R Core Team (2018). *R: A Language and Environment for Statistical Computing.* Vienna: R Foundation for Statistical Computing.

[B61] Rescorla L. Dahlsgaard K. Roberts J. (2000). Late-talking toddlers: MLU and IPSyn outcomes at 3;0 and 4;0. *J. Child Lang.* 27 643–664. 10.1017/s0305000900004232 11089342

[B62] Schauwers K. Gillis S. Daemers K. De Beukelaer C. Govaerts P. J. (2004). Cochlear implantation between 5 and 20 months of age: the onset of babbling and the audiologic outcome. *Otol. Neurotol.* 25 263–270. 10.1097/00129492-200405000-00011 15129103

[B63] Schramm B. Keilmann A. Brachmaier J. (2010). Canonical babbling and early hearing and language development of normal hearing children and children with cochlear implants. *Cochlear Implant Int.* 11 375–378. 10.1179/146701010X12671177990073 21756653

[B64] Spencer P. E. (2004). Individual differences in language performance after cochlear implantation at one to three years of age: child, family, and linguistic factors. *J. Deaf Stu. Deaf Educ.* 9 395–412. 10.1093/deafed/enh033 15314014

[B65] Squire L. R. Zola S. M. (1996). Structure and function of declarative and nondeclarative memory systems. *Proc. Natl Acad. Sci. U.S.A.* 93 13515–13522. 10.1073/pnas.93.24.13515 8942965 PMC33639

[B66] Stoel-Gammon C. (1988). Prelinguistic vocalizations of hearing impaired and normally hearing subjects. *J. Speech Hear. Disord.* 53 302–315. 10.1044/jshd.5303.302 3398483

[B67] Stoel-Gammon C. (1989). Prespeech and early speech development of two late talkers. *First Lang.* 9 207–223. 10.1177/014272378900900607

[B68] Stoel-Gammon C. (2011). Relationships between lexical and phonological development in young children. *J. Child Lang.* 38 1–34. 10.1017/s0305000910000425 20950495

[B69] Svirsky M. A. Robbins A. Kirk K. Pisoni D. Miyamoto R. (2000). Language development in profoundly deaf children with cochlear implants. *Psychol. Sci.* 11 153–157. 10.1111/1467-9280.00231 11273423 PMC3429133

[B70] Svirsky M. A. Teoh S. W. Neuburger H. (2004). Development of language and speech perception in congenitally, profoundly deaf children as a function of age at cochlear implantation. *Audiol. Neuro Otol.* 9 224–233. 10.1159/000078392 15205550

[B71] Szagun G. Schramm S. A. (2016). Sources of variability in language development of children with cochlear implants: age at implantation, parental language, and early features of children’s language construction. *J. Child Lang.* 43 505–536. 10.1017/S0305000915000641 26597734

[B72] Thelen E. (1981). Rhythmical behavior in infancy: an ethological perspective. *Dev. Psychol.* 17 237–257. 10.1037/0012-1649.17.3.237

[B73] Tomblin J. B. Barker B. A. Spencer L. J. Zhang X. Gantz B. J. (2005). The Effect of age at cochlear implant initial stimulation on expressive language growth in infants and toddlers. *J. Speech Lang. Hear. R.* 48 853–867. 10.1044/1092-4388(2005/059) PMC3209960 16378478

[B74] Vihman M. M. (1996). *Phonological Development: The Origin of Language in the Child.* Oxford: Wiley-Blackwell.

[B75] Vihman M. M. (2014). *Phonological Development: The First Two Years.* Oxford: Wiley-Blackwell.

[B76] Volling B. L. Cabrera N. J. Feinberg M. E. Jones D. E. McDaniel B. T. Liu S. (2019). Advancing research and measurement on fathering and children’s development. *Monogr. Soc. Res. Child* 84 7–160. 10.1111/mono.12404 31034620

[B77] Walker E. A. Bass-Ringdahl S. (2008). Babbling complexity and its relationship to speech and language outcomes in children with cochlear implants. *Otol. Neurotol.* 29 225–229. 10.1097/mao.0b013e31815f6673 18165791

[B78] Westermann G. Miranda E. R. (2004). A new model of sensorimotor coupling in the development of speech. *Brain Lang.* 89 393–400. 10.1016/S0093-934X(03)00345-6 15068923

[B79] Whitehurst G. J. Smith M. Fischel J. E. Arnold D. S. Lonigan C. J. (1991). The continuity of babble and speech in children with specific expressive language delay. *J. Speech Hear. R.* 34 1121–1129. 10.1044/jshr.3405.1121 1749242

